# Safety and Efficacy of* Ferula asafoetida* in Functional Dyspepsia: A Randomized, Double-Blinded, Placebo-Controlled Study

**DOI:** 10.1155/2018/4813601

**Published:** 2018-08-26

**Authors:** K. N. Mala, Jestin Thomas, Das S. Syam, Balu Maliakel, I. M. Krishnakumar

**Affiliations:** ^1^Sri Rama Hospital, Fort road, Doddaballapur, Bangalore, India; ^2^Leads Clinical Research & Bio Services Pvt. Ltd., Bangalore, India; ^3^R&D Centre, Akay Flavours & Aromatics Pvt. Ltd., Kerala, India

## Abstract

Despite the availability of various synthetic drugs for the treatment of functional dyspepsia (FD), the side effects and their cost have always created a great interest in the search for novel natural alternatives for the management of gut disorders. The present contribution reports the safety and efficacy of the kitchen spice asafoetida (*Ferula asafoetida*) in FD for the first time. In the double-blinded, placebo-controlled study, 43 subjects diagnosed to have moderate to severe discomforts of nonulcer FD were randomized to receive hard-shell capsules (250 mg × 2/day) of either placebo (n=22) or a food-grade formulation of asafoetida (Asafin) (n=21) for 30 days. When evaluated by a set of validated indexing tools (GSRS, GDSS, and NDI), almost 81% in the Asafin group showed significant (*p < 0.01*) improvement in the overall score and quality of life as compared to the placebo. At the end of the study, 66% of subjects in the Asafin group remained symptoms-free. Although the symptoms score improved significantly in both the groups (from -5.67 to -25.29 in Asafin group versus -1.55 to -6.0 in the placebo;* p ≤ 0.001*), the relative percentage of subjects in the Asafin group with more than 80% reduction in various symptoms were: bloating (58%), appetite (69%), postprandial fullness (74%) motion sickness (75%), and digestion (77%) as compared to less than 10% nonspecific improvement in the placebo group. All the subjects remained safe with no adverse events or variations in haematological and biochemical parameters. The study was registered at http://ctri.nic.in/ (CTRI/2018/ 01/011149).

## 1. Introduction

Functional dyspepsia (FD) is one of the most common functional gastrointestinal disorders (FGIDs) that have been shown to affect the gastroduodenal region of the gastrointestinal (GI) tract with no identifiable structural lesions [1]. FD is normally characterized with discomforts such as bloating, early satiety, postprandial fullness, belching, heart burn, indigestion, and epigastric pain leading to the poor quality of life [2, 3]. Though it does not cause mortality, FD is a costly gastroenterology practice which is highly prevalent in all populations with a relatively increased rate of incidence among females [3, 4]. In the United States, the prevalence rate has been found to be more than 40% with an expense of $ 18.4 billion towards the average healthcare cost in 2009 [5]. An epidemiological study in Europe has also demonstrated 40% dyspepsia prevalence with multiple medical visits and medication for more than 50% of the individuals [6].

A number of factors such as duodenal eosinophilia, psychological distress, gastroduodenal dysfunction,* Helicobacter pylori *infection, smoking, alcoholism, chronic usage of nonsteroidal anti-inflammatory drugs (NSAIDs), and sedentary life style have very often been delineated to the pathogenesis of FD [1, 7–9]. Despite varying results of controlled clinical trials, the current pharmacologic treatment modalities include the use of* Helicobacter pylori *inhibitors, acid suppressants, proton pump inhibitors, antidepressants, antacids, and prokinetics [10–13]. Gastric acid suppression and enhancement of gastrointestinal motility are the underlying mechanisms of most of these drugs. However, these treatments are very often complicated by the limited response, high cost, and side effects [14]. For instance, Cisapride (*cis*-4-amino-5-chloro-N-{1-[3-(4-fluorophenoxy)propyl]-3-methoxy-4-piperidinyl}-2-methoxy benzamide), a prokinetic drug that has recently been shown to be efficient upon several randomized, controlled trials and meta-analyses, was withdrawn in most countries due to cardiac side effects [15]. Thus, there has always been a great interest on natural agents, especially those from food components such as fruits, vegetables, and spices, for either the treatment or the maintenance of gut health [16, 17].

Extracts of artichoke, liquorice, ginger, black cumin, and basil have already been investigated for their effects on FD [18–22]. In the present contribution, a novel food-grade formulation of the oleo-gum-resin of asafoetida (*hereinafter referred to as “Asafin”*) has been investigated (double-blinded, placebo-controlled, and randomized study) for its efficacy and safety on subjects diagnosed to have FD.* Ferula asafoetida* (Umbelliferae) is an herbaceous wild plant native to the mountains of Afghanistan and its latex called* asafoetida oleo-gum-resin* is a popular kitchen spice and traditional medicine for gut health [23]. Earlier studies have suggested various mechanisms for the gastrointestinal smooth muscle relaxing effect of asafoetida. These include the blocking action on excitatory pathways such as cholinergic [24], histaminergic [25], or mimicking the action of inhibitory systems such as adrenergic [26], purinergic [27], GABAergic [28], and/or nitric oxide [29]. Pharmacological studies have also demonstrated the antioxidant, antiviral, antimicrobial, antidiabetic, and gastroprotective activities of asafoetida [30]. However, no clinical evaluations have so far been reported on the therapeutic efficacy of asafoetida gum against any of the health disorders. Gummy nature, pungency, and the unpleasant odour due to relatively high levels of essential oil rich in sulphur compounds [10 to 20% (w/v)] have been identified as the main physicochemical and organoleptic hurdles associated with the wide spread use of asafoetida gum. Asafin used in the present study is a formulation of asafoetida oleo-gum-resin encapsulated with soluble dietary fibre (galactomannans), isolated from fenugreek seeds (*Trigonella foenum gracum*) [31]. Fenugreek galactomannans are good prebiotics with hypolipidemic, hypoglycemic, and gastroprotective effects [32]. High viscosity, gel formation, encapsulation efficiency, mucoadhesive nature, and gastroretentive property of fenugreek galactomannans have already been established as a good vehicle for the formulation of oral delivery solid dosage forms of bioactive molecules [33].

## 2. Materials and Methods

### 2.1. Subjects and Study Design

The study was conducted at M/s Sri Rama Hospital, Bangalore, India, under the supervision of a qualified medical doctor, following a randomized, double-blinded, placebo-controlled design. The study was in strict accordance with the clinical research guidelines of Government of India following the protocol approved (dated 15/07/2016) by the registered ethical committee (Reg. No. ECR/184/Int/KA/2014) and was retrospectively registered in clinical trial registry of India at http://ctri.nic.in/ (CTRI/ 2018/ 01/011149). The subjects were selected from the out patients who approached the doctor for medical consultation on gastrointestinal disorders. Those subjects who have been characterized to have functional dyspepsia based on Rome III Diagnostic Criteria were enrolled in the study with a written consent [34]. According to Rome III criteria, subjects with no suspected structural diseases such as gastric ulcers, but characterized to have symptoms of bothersome postprandial fullness, early satiation, epigastric pain, and epigastric burning, were identified to have FD. Details of inclusion and exclusion criteria followed in the study were given in Table 1. The power used to calculate sample size is 80% and got a minimum sample size of 40 required for the study. A total of 60 subjects (aged between 25 and 55 years) were enrolled and provided with a unique three-digit randomization code.

**Figure 1:**
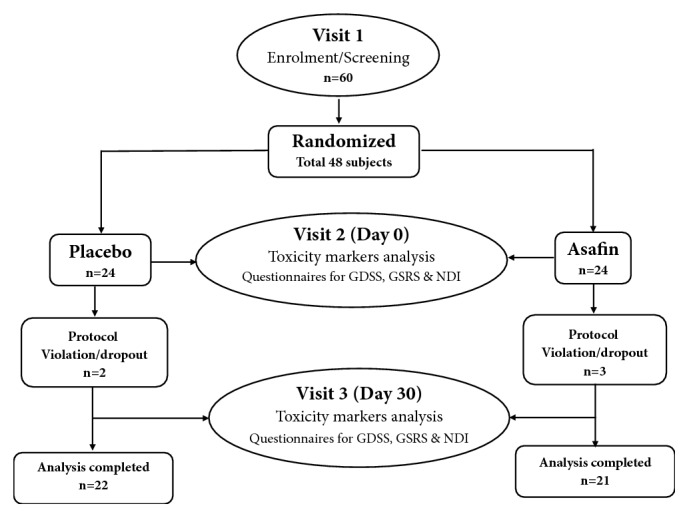
Cohort diagram showing study design.

The protocol used in the present study was depicted in Figure 1. Total study period was 30 days. Subjects were requested to visit their designated study site on three different occasions, namely, visit 1- screening and enrolment; visit 2- baseline/randomization (Day 0); and visit 3- end of study (EOS) (Day 30). The baseline characteristics are provided in Table 3. A physical examination and laboratory tests (including red-cell counts, packed cell volume, mean corpuscular volume, liver-function tests and renal function tests), were performed during visit 1 (Screening) and on visit 3 (Day 30). During visit 1, tweleve subjects refused to participate in the study. Sequentially numbered airtight high density polyethylene containers each containing 90 numbers of 250 mg capsules of either Asafin or placebo were provided on visit 1 (Day 0) and instructed to take one capsule before the breakfast and another one before dinner (250 mg × 2/day). The degree of adherence of the subjects was assessed by “count pill” strategy. Asafin has the characteristic smell of asafoetida oleo-gum-resin and the placebo was MCC. In order to have the asafoetida oleo-gum-resin like smell, MCC was plated with 0.01% of asafoetida oleo-gum-resin. Thus, each bottle (Asafin and placebo) of capsules was managed to have identical smell. Blinding efficacy was assessed by giving a chance to guess the group to which each subject was assigned to ensure the smell identification is possible or not. Subjects were monitored on a weekly basis through regular telephonic follow-ups and short message services. Asafoetida gum content in 250 mg Asafin was standardized to 90±5 mg.

### 2.2. General

The proprietary formulation of asafoetida-gum-resin (patent pending and registered formulation as ‘Asafin®') was obtained from M/s Akay Flavours & Aromatics Ltd., Cochin, India, along with a detailed certificate of analysis indicating its relative composition of asafoetida gum, dietary fibre, volatile oil, and ferulic acid content [31]. Safety parameters including pesticides, microbial counts, mycotoxins, and heavy metal content of both Asafin and placebo (microcrystalline cellulose, MCC) were analyzed as per USFDA requirements for dietary supplements [35]. Asafin was prepared from Iranian asafoetida and voucher specimen (AK-ASF-01) was deposited at the Herbarium of M/s Akay Flavours & Aromatics Ltd., Cochin, India. Safety of Asafin was assessed by lethal dose (LD_50_) and subacute repeated dose 28-day toxicity study on Wistar rats [31]. Volatile oil content was measured using a standardized and approved method of American Spice Trade Association (ASTA, 1997) [36]. Ferulic acid standard was obtained from Sigma-Aldrich, Bangalore, India, and estimated by a high performance liquid chromatography (HPLC). Shimadzu model LC 20 AT, with an M20A photo diode array (PDA) detector (Shimadzu Analytical India Pvt. Ltd., Mumbai, India), fitted with a reverse phase C18 column (250 × 4.6 mm, 3 *μ*m) (Phenomenex, Hyderabad, India) was used for analysis. Water with 10% acetic acid and acetonitrile with 20% acetic acid were as the mobile phases and monitored at 319 nm. Scanning electron micrograph was performed on SEM Jeol 6390 LA equipment (JEOL Ltd., Tokyo, Japan).

### 2.3. Efficacy Determination

The efficacy end points were primarily assessed by three validated questionnaires; Gastrointestinal Symptom Rating Scale (GSRS) [37, 38], Glasgow Dyspepsia Severity Score (GDSS) [39, 40], and Nepean Dyspepsia Index (NDI) [41, 42]. The questionnaires were provided at visit 2 (Day 0) and visit 3 (Day 3) of the study. GSRS consists of a seven-point graded Likert-type scale where “1” represents the absence of troublesome symptoms and “7” represents the most troublesome symptoms [43]. GDSS provides a global measurement of the severity of dyspepsia using seven questions from seven different categories with regard to upper gastrointestinal symptoms. The present study employed a modified version of GDSS where 0 was the minimum possible score and 20 was the maximum score, in the ascending order of discomfort level [39]. NDI is one of the most recent disease-specific indexes for dyspepsia, which measures symptoms and health-related quality of life [44]. It originally contained 42 items designed to measure the impact of FD on a subject's ability to engage in relevant aspects of their life [41]. The present study employed the short form (NDI-SF) of NDI having 10 items, with a 5-point Likert scale ranging from 0 (not at all or not applicable) to 4 (extremely applicable) [45].

### 2.4. Safety Evaluation

The primary safety and tolerability of Asafin at the present dosage of (250 mg × 2/day) was evaluated by specifically collecting the individual data regarding any adverse reactions, clinical changes, or discomforts. Haematological and biochemical parameters at the beginning and at the end of the study were also conducted as a measure of safety. Red blood cell (RBC) count, haemoglobin level (Hb), packed cell volume (PCV), mean corpuscular volume (MCV), mean corpuscular haemoglobin (MCH), and mean corpuscular haemoglobin concentration (MCHC) were determined using a haematology analyzer (Model-Diatron, Wien, Austria). Plasma was separated by centrifugation at 11,950g for 10 min at 4°C and stored for a maximum of two days at −20°C for biochemical analysis. Biochemical parameters such as glutamate oxaloacetate transaminase (SGOT), glutamate pyruvate transaminase (SGPT), and alkaline phosphatase (ALP) levels and serum creatinine were analyzed by following the assay kits provided by M/s Agappe Diagnostics Pvt. Ltd., Bangalore, India. Random blood sugar levels were analyzed by ACCU-CHEK active glucose test strips and glucose meter (Roche Diagnostic GmbH, Mannheim, Germany).

### 2.5. Statistical Analysis

Statistical analyses were carried out using the Statistical Package for Social Science (SPSS Inc. Chicago, IL, USA) version 17.0. Total sample size at the end of study was 43. The efficacy end points included the comparison of data at the baseline and at the end of the study (within group comparison) and also the comparison with the placebo group (between group comparisons). Within group comparison was done with paired sample* t*-test and between group comparisons were done using independent sample* t*-test and* p ≤ 0.05* was considered to be significant. The results were presented as mean ± SEM. 95% CI values are also provided. Mean difference was compared by using independent sample* t*-test.

## 3. Results

**Figure 2:**
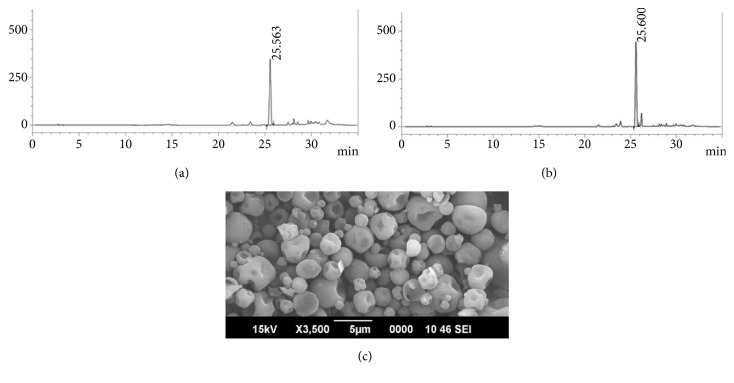
(a) HPLC profile of Asafoetida raw material; (b) HPLC profile of Asafin; (c) SEM photograph of Asafin indicating the microencapsulation with the soluble dietary fibre (galactomannans) from fenugreek.

Iranian asafoetida oleo-gum-resin was employed for the preparation of Asafin used in the present study. Gel-phase dispersion followed by microencapsulation of asafoetida oleo-gum-resin on fenugreek soluble fibre (galactomannans) matrix provided water soluble free-flowing granules of Asafin with particle size of around 250±50 *μ*m suitable for the manufacture of capsules, tablets, and softgels. Water based preparation process with no organic solvents and synthetic chemicals provided a unique green formulation suitable for food and nutraceutical applications. HPLC finger printing revealed the stability of Asafin as compared to asafoetida gum with 1.5% of ferulic acid content (Figures 2(a) and 2(b)). Stable encapsulation of asafoetida gum within the fenugreek soluble dietary fibre was clear from the SEM photographs (Figure 2(c)). Upon detailed analysis of the nutritional composition and food safety, parameters demonstrated the adherence of Asafin to international regulation for use as dietary supplements (Table 2).

### 3.1. Effect of Asafin on FD Symptoms and Severity Score

**Figure 3:**
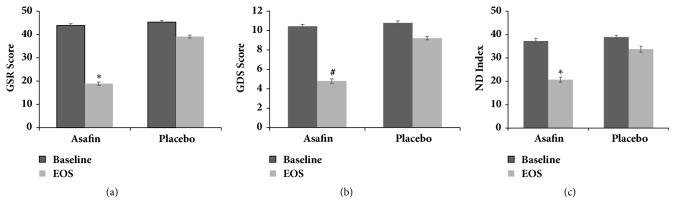
(a) Gastrointestinal Symptom Rating Scale (GSRS); (b) Glasgow Dyspepsia Severity Score (GDSS); (c) Nepean Dyspepsia Index-Short Form (NDI-SF). Values are expressed as mean ± SEM. The values not sharing a superscript significantly differ at p ≤ 0.001.

The present study employed FD symptoms rating scales (GSRS, GDSS, and NDI) to evaluate the efficacy of Asafin supplementation on individual dyspepsia symptoms and overall severity score as compared to placebo and baseline. A significant (p≤ 0.001) reduction (51.6%) in overall symptoms severity score was observed when GSRS scores of Asafin group were compared with placebo (Table 4). Within group comparison of GSRS scores with that at the end of the study period using paired sample* t*-test showed a significant reduction in Asafin group (95% confidence interval: −25.29; 44.19±0.85 to 18.90±0.67; p ≤0.001) as compared to the placebo (95% confidence interval: −6; 45.09±0.72 to 39.09±0.67; p ≤0.001) (Figure 3(a)). Between group comparison showed no significant difference between baseline values of placebo with Asafin treated group but showed significant difference in the end of study values when compared to placebo (Table 4; 95% CI 2.61, 54.98).

Inner group comparison of the baseline values with those at the end of the study showed a significant reduction of 54% in GDSS score when treated with Asafin (95% confidence interval: from 10.45±0.21 to 4.80±0.17; p ≤ 0.001), as compared to the 14% reduction in placebo (95% confidence interval: from 10.79±0.20 to 9.22±0.25;* p > 0.05*) (Figure 3(b)). Intergroup comparison also showed a significant reduction in GDSS score of the Asafin group (*p ≤0.001*; 47%), as compared to the placebo (95% confidence interval: placebo 9.22±0.23; Asafin 4.80±0.17) (Table 4). Almost 69% subjects in the Asafin group reported significant reduction in the frequency of FD symptoms and 87% reported no usage of any synthetic drugs during the course of the study. Between group comparison showed no significant difference between baseline values of placebo with Asafin treated group but showed significant difference in the end of study values when compared to placebo (Table 4; 95% CI 1.70, 28.02).

Analysis of the NDI scores also confirmed a significant (*p ≤0.001*) reduction (38.67%) on overall symptoms in Asafin group as compared to the placebo (Table 4). Inner group comparison with respect to the baseline and end of the study demonstrated a relative difference of -16.57 (from 37.28±0.71 to 20.71±1.09) in Asafin group and -5.32 (from 39.09±1.28 to 33.77±1.07) in case of placebo (Figure 3(c)). Intergroup comparison indicated a difference of -13.06 in NDI score when the scores of the Asafin with placebo group at the end of the study were compared (95% confidence interval: 37.28±0.71 to 20.71±1.09). Between group comparison showed no significant difference between baseline values of placebo with Asafin treated group but showed significant difference in end of study values when compared to placebo (Table 4; 95% CI 2.06, 35.08).

A comparison of all the three symptoms rating scores (GSRS, GDSS, and NDI) indicated similar baseline values for both the Asafin and placebo groups, with no significant differences (Table 4). But, at the end of the study, Asafin group showed significant reduction in all the three scores (81% of subjects), indicating its primary efficacy against FD (Figure S1). When the individual symptoms were monitored, the most beneficial effects were observed in bloating, postprandial fullness, early satiety, constipation, and indigestion, which were found to be significantly reduced from second week of the study period onwards. The percentage of people who reported more than 50% reduction in their discomfort levels were given in Table S2. Upon completion of the study, the relative percentage of subjects who reported more than 80% reduction were in bloating (58%), appetite (69%), postprandial fullness (74%), motion sickness (75%), and digestion (77%) for Asafin treated group as compared to less than 10% nonspecific improvement reported in the placebo group. It is also noted that 66% of subjects in Asafin group remained symptoms-free and 75% of the subjects reported an improvement in their ability and/or interest to carry out daily works with more stability and focus of mind due to the reduction in the frequency of FD symptoms. Almost 87% reported no usage of any synthetic drugs during the course of the study.

### 3.2. Safety Evaluation Studies

Within group comparison and between groups comparison results of haematological and biochemical analysis were given in Table 5. It was observed that the treatment of both Asafin and placebo did not produce any significant (*p > 0.05*) changes on haemoglobin content, RBC count, PCV, MCV, MCH, and MCHC concentrations as compared to the baseline values. Biochemical parameters such as liver-function markers (SGOT, SGPT, and ALP) and the renal function marker, (serum creatinine) also remained within the normal range upon treatment with both Asafin and placebo.

## 4. Discussion

Good digestion is an essential component for a good quality of life and well-being, since the digestive system is responsible for the retention of the nutrients and elimination of the waste. A variety of reasons including, but not limited to, sedentary life style, lack of exercise, food intake without appetite, irregularity in breakfast, chronic alcoholism, smoking, stress/anxiety, and oily food were found to induce gastrointestinal disorders, in addition to the pathogenesis of some diseases. FD is one of the most common digestive upset and its prevalence has been reported to vary from 11 to 30% among adult population [6]. Since FD is not life-threatening, it is not surprising that the primary choice of treatment option is the alternative therapies with natural agents widely used in various traditional systems of medicine. Though a number of botanical supplements are currently available for gut health, the majority of them have failed to provide satisfactory efficacy. Unstandardized solvent extraction techniques followed by harsh conditions of formulations of botanical extracts may be the main reasons for the lack of efficacy of botanical extracts. In the present study, a food-grade oleo-gum-resin of asafoetida, a GRAS-listed (Generally Recognised as Safe) kitchen spice widely used in India and other Asian countries, was employed. Considering the unpleasant organoleptic characteristics and sticky paste-like form of the natural asafoetida gum, a green formulation (Asafin) employing the fenugreek soluble dietary fibre (galactomannans) and water was used in the present study. Water based process of impregnation of asafoetida oleo-gum-resin into the soluble dietary fibre matrix under mild conditions of temperature under vacuum to provide free-flowing water soluble powder of Asafin and its characterization, stability, and controlled-release properties has recently been published [31]. Though the meaning of the Latin word* “assa-foetida” *itself is* “Carrier of bad smell”* with its common name as “*Devils*'* dunk”* indicating the degree of unpleasant flavour characteristics of asafoetida oleo-gum-resin, uniform impregnation of the lipophilic gum into the hydrophilic matrix of the dietary fibre was found to provide taste and odour masked Asafin particles suitable for dietary applications.

Except the recent* in vivo* study on the gastroprotective effect of asafoetida gum by Babaeian et al., no detailed reports (*in vitro *or* in vivo*) have been available on the gastroprotective effect and mechanism of action of asafoetida. Recently, we had reported the antioxidant, anti-inflammatory, and antiulcerogenic activities of Asafin on alcohol-induced ulcer model of rats [31]. While the antioxidant and anti-inflammatory activities were marginal, Asafin exhibited antiulcerogenic activity and safety with a significant enhancement in the gastric mucosa production [31]. The present randomized, placebo-controlled, and double-blinded pilot study employed Asafin containing about 42% (w/w) asafoetida-gum-oleo-resin formulated with debitterised fenugreek powder rich in soluble dietary fibre, so that each 250 mg capsules of Asafin provided 90±5 mg of asafoetida-gum. When supplementing two capsules per day (180±10 mg of asafoetida gum/day), the subjects with moderate to severe FD symptoms were found to have significant reduction in the gut disorders with an improvement in the quality of life as compared to placebo. While the FD symptoms were rated with well-validated symptoms scores scales (GSRS, GDSS, and NDI), 69% of the subjects reported to have significant reduction in symptoms scores with 87% of the subjects reporting no usage of synthetic drugs during the study period, as compared to the placebo group where 61% of the subjects reported the repeated use of synthetic drugs during the course of the study.

The GSRS is one of the most established and responsive disease-specific instrument with five symptom clusters depicting reflux, abdominal pain, indigestion, diarrhoea, and constipation. The seven-point graded Likert-type scale in GSRS with ascending order of severity of the symptoms has widely been used in FD studies [46, 47]. In the present study, Asafin treatment for 30 days showed significant (p ≤ 0.05) improvement in both inner and intergroup comparisons as compared to the placebo. The improvements in symptom scores and severity score were also clear from the other two scales (GDSS and NDI) used in the present study. GDSS has been well-validated as a global measurement of the severity of dyspepsia in patients with upper gastrointestinal disorders and has been shown to provide valuable responses on treatment [39]. A large number of studies have been globally reported in validating GDSS and used widely to evaluate the treatment efficacy [40, 48]. The fact that the present study provided highly significant beneficial response (p ≤0.001) with more than 50% reduction in severity scores for more than 80% of subjects in the Asafin group demonstrates the efficacy of Asafin over the placebo group with only less than 13% improvement in the symptoms score. NDI is yet another most commonly used analysis tool for FD specifically to measure the symptoms and health-related quality of life [38]. NDI index was validated by Nkurunziza et al. and has been widely used in many recent studies [45, 49, 50]. In the present study, NDI scores were significantly reduced (47%) in Asafin treated group in comparison with the baseline values, whereas no significant change was observed in placebo.

It was also observed that almost 62% of subjects in the Asafin group showed >50% improvement in quality of life with a significant reduction in individual FD symptoms. Bloating, postprandial fullness, ability to eat, constipation, and digestion were the most significantly affected symptoms. Further, Asafin does not show any adverse effects or clinically significant changes in either haematological or biochemical parameters, indicating its safety and tolerance at the dosage of 250 mg × 2/day for 30 days. Thus, the present study demonstrated the safety and efficacy of asafoetida oleo-gum-resin and its gut health potential for the first time, paving the way forward with larger multicentred trials. However, relatively small number of patients and lack of endoscopic characterization of the patients remain as the drawbacks of the present study. Further studies involving 30 to 60 days of supplementation with at least one month of observation period and intervention on populations where asafoetida is not a regular part of the diet, such as in the West, will be of great interest.

## 5. Conclusion

Management of functional dyspepsia has always been a challenge due to the side effects and the cost associated with the synthetic drugs. The present double-blinded, placebo-controlled, randomized study on 43 subjects characterized with functional dyspepsia demonstrated the safety and efficacy of* Ferula asafoetida *oleo-gum-resin for the first time, when supplemented as a food-grade formulation with fenugreek dietary fibre (Asafin) for 30 days at a dosage of 250 mg ×2/day, containing around 36% (w/w) of asafoetida gum. While 81% of the subjects treated with Asafin showed significant improvement in overall symptoms score, 66% of the subjects remained symptoms-free at the end of the study. Almost 67% of the subjects in the Asafin group improved the quality of their life with better interest and focus on their daily works from the second week onwards with a significant improvement in bloating, postprandial fullness, food intake, heart burn, constipation, and digestion with no side effects or adverse events as demonstrated by the blood analysis.

## Figures and Tables

**Table 1:** Inclusion and exclusion criteria used for subject's eligibility.

**Inclusion Criteria**
(i) Age 25-60 years ( both inclusive)
(ii) Male and female subjects
(iii) Fulfilling Rome III Diagnostic Criteria for Functional Dyspepsia
(iv) Must meet the criteria for 3 months and must begin experiencing symptoms for at least 6 months before diagnosis.
(v) Subject willing to give written informed consent

**Exclusion Criteria**

(i) History of Peptic ulcer, Gastro-oesophageal Reflux Disease, Gastro intestinal surgery or any other clinically significant gastrointestinal disease
(ii) Psychiatric illness
(iii) Pregnant or lactating women
(iv) History of congestive heart failure or uncontrolled hypertension
(v) Subjects with abnormal haematological or biochemical parameters
(vi) Subjects who have taken antibiotics or any other drugs in last 2 weeks whose primary site of action is in the Gastrointestinal tract
(vii) Any condition that in opinion of the investigator, does not justify the subjects' participation in the study

**Table 2:** Physicochemical characteristics of Asafin.

**Parameters**	**Test results**
Colour and Appearance	Off-White free flowing powder
Odour	Mild characteristic
Solubility	Soluble in water upon homogenisation
Moisture (%)	4.2
Bulk density (g mL^−1^)	0.56
Volatile oil content (%)^*∗*^	3.4

**Nutrition Facts/100 g**°	
Carbohydrates	82.0 g
Proteins	6.1g
Dietary fibre	6.1g
Fat (hexane solubles)	3.2g
Energy	381.2kcal

**Microbiology** ^**#**^	
Total plate count	< 3000 cfu g^−1^
Yeast & mould	< 100 cfu g^−1^
E. coli	Absent
Coliforms	< 3 MPN g^−1^
Salmonella	Absent

**Heavy Metals** ^**!**^	
Lead	< 0.5 ppm
Mercury	< 0.1 ppm
Cadmium	< 0.5 ppm
Arsenic	< 1 ppm

^**∗**^ Method: ASTA method no. 5.2, 4th Ed; 2010.

°Method: AOAC Pearson's composition and analysis of food 19th Ed; 1991.

^**#**^Methods: FDA BAM Ch 3-5, 18th Ed; 2011.

^**!**^Method: AAS method AOAC,18th Ed; 2005.

**Table 3:** Baseline characteristics of study subjects.

**Characteristics**	**Baseline** **(N=43)**
Age (years)	29.46 ± 6.24

Height (Cm)	158.7 ± 5.3

Weight (kg)	60.5 ± 6.4

BMI (kg/m^2^)	24.22 ± 2.37

Systolic BP (mmHg)	114.07 ± 7.34

Diastolic BP (mmHg)	70.20 ± 8.74

Pulse	70.13 ± 6.73

Data expressed as mean ± SD.

**Table 4:** Statistical comparison of dyspepsia indexing scores.

**Name of Tool**	**Intergroup comparison based on ** **independent sample t-test**			**Inner group comparison ** **based on paired sample t-test**			**95**%** CI Values**	**Mean difference comparison of variables using independent sample t-test**	**p-value**
	**Time of Observation**	**Placebo**	**Asafin**	**Groups**	**Baseline**	**End of Study**			
**GSRS**	Baseline	45.33±0.77	43.91±0.76	Placebo	45.09±0.72	39.09±0.07	2.61	6.00±0.33	
	End of study	39.09±0.67	18.90±0.67^#^	Asafin	44.19±0.85	18.40±0.67^#^	54.98	25.28±0.89	
**GDSS**	Baseline	10.79±0.20	10.45±0.21	Placebo	10.77±0.22	9.22±0.23	1.70	1.90±0.20	p<0.001
	End of study	9.22±0.23	4.80±0.17^*∗*^	Asafin	10.47±0.22	4.80±0.17^*∗*^	28.02	5.66±0.24	
**NDI**	Baseline	38.95±1.20	37.25±0.68	Placebo	39.09±1.28	33.77±1.07	2.06	5.38±0.46	
	End of study	33.77±1.07	20.71±1.09^+^	Asafin	37.28±0.71	20.71±1.09^+^	35.08	16.57±1.16	

Statistical comparison of GSRS, Gastrointestinal Symptom Rating Scale; GDSS, Glasgow Dyspepsia Severity Score; and NDI, Nepean Dyspepsia Index values of Placebo with Asafin using paired sample *t*-test (within group) and independent sample *t*-test (between groups). The results are given as mean ± SEM. Values having a superscript significantly differ from its placebo at p<0.001. 95% CI values and mean difference comparison are also given.

**Table 5:** Results of safety evaluation studies.

**Parameters**	**Intergroup comparison based ** **on independent sample t-test**			**Inner group comparison ** **based on paired sample t-test**			**Mean difference comparison of variables using independent sample t-test**	
	**Time of Observation**	**Placebo**	**Asafin**	**Groups**	**Baseline**	**End of Study**	**Mean difference**	**p-value**
**Hb **(g/dl)	Baseline End of study	13.25±0.49 13.20±0.47	13.45±0.46 13.78±0.49	Placebo Asafin	13.26±0.17 13.45±0.15	13.20±0.82 13.78±0.20	0.06±0.04 0.33±0.05	0.219

**RBC Count **(million/*µ*L)	Baseline End of study	4.24±0.15 4.25±0.16	4.26±0.15 4.25±0.15	Placebo Asafin	4.25±0.37 4.26±0.06	4.25±0.33 4.26±0.04	0.009±0.02 0.003±0.02	0.940

**Packed cell volume **(%)	Baseline End of study	38.30±0.36 38.82±0.36	39.22±0.45 39.88±0.40	Placebo Asafin	38.31±0.42 39.22±0.50	38.82±0.38 39.88±0.47	0.50±0.13 0.66±0.18	0.261

**Mean corpuscular volume **(fl/red cell)	Baseline End of study	90.60±0.22 91.02±0.25	91.82±0.24 92.92±0.23	Placebo Asafin	90.61±0.84 91.83±0.56	91.02±0.88 92.93±0.47	0.40±0.12 1.09±0.25	0.067

**Mean corpuscular haemoglobin **(pg/cell)	Baseline End of study	31.32±0.13 31.25±0.48	31.52±0.27 31.71±0.19	Placebo Asafin	31.33±0.24 31.52±0.20	31.25±0.23 31.71±0.13	0.07±0.22 0.09±0.17	0.813

**Mean corpuscular haemoglobin Concentration **(g/dl)	Baseline End of study	34.42±0.25 34.09±0.22	34.32±0.18 34.24±0.17	Placebo Asafin	34.42±0.18 34.33±0.20	34.09±0.21 34.24±0.23	0.33±0.05 0.08±0.13	0.477

**SGOT **(IU/L)	Baseline End of study	25.46±0.93 26.28±0.93	27.18±0.93 25.65±0.90	Placebo Asafin	25.46±1.00 27.18±1.20	26.28±0.75 25.66±1.07	0.82±0.15 1.52±0.18	0.771

**SGPT **(IU/L)	Baseline End of study	33.27±1.22 38.71±0.93	35.14±1.21 33.65±1.17	Placebo Asafin	33.28±0.94 35.14±1.74	38.71±0.96 33.66±1.39	5.43±0.25 1.48±0.11	0.001

**ALP **(IU/L)	Baseline End of study	85.32±2.97 95.53±2.33	89.92±2.10 95.11±2.32	Placebo Asafin	85.32±2.12 89.92±2.50	95.53±2.07 95.11±2.57	10.20±0.52 5.19±0.30	0.027

**S. Creatinine **(mg/dl)	Baseline End of study	0.77±0.02 0.81±0.02	0.82±0.02 0.76±0.02	Placebo Asafin	0.78±0.02 0.81±0.02	0.81±0.02 0.77±0.02	0.02±0.001 0.061±0.01	0.006

Values are expressed as mean ± SEM. Hb: haemoglobin, RBC: red blood cells, SGOT: serum glutamic oxaloacetic transaminase, SGPT: serum glutamic pyruvic transaminase, ALP: alkaline phosphatase and S.Creatinine: serum creatinine. Within group comparison was done using paired sample *t*-test and between group comparisons were done with independent sample *t*-test. Mean difference comparison of variables was performed by independent sample *t*-test. P value is also provided.

## Data Availability

The data used to support the findings of this study are available from the corresponding author upon request.

## Supplementary Materials

Supplementary Materials Supplementary material 1: reduction in FD Symptom indexing Scores. Supplementary material 2: comparison of individual symptom scores.
