# Supplementary material for: Safety and Efficacy of Ferula asafoetida in Functional Dyspepsia: A Randomized, Double-Blinded, Placebo-Controlled Study
Source: Evid Based Complement Alternat Med. 2018 Aug 26;2018:4813601. doi: 10.1155/2018/4813601 (PMC6129344; doi:10.1155/2018/4813601)
Supplement: Supplementary Materials — Supplementary material 1: reduction in FD Symptom indexing Scores. Supplementary material 2: comparison of individual symptom scores. [file 4813601.f1.docx]

**SUPPLEMENTARY FILE**

**Safety and efficacy of *Ferula asafoetida* in functional dyspepsia – a randomized, double-blinded, placebo controlled study**

**Mala KN^a^, Jestin Thomas^b^, Syam Das S^c^, Balu Maliakel^c^ & Krishnakumar IM^c^***

*Corresponding author

Krishnakumar IM, Ph.D, Chief Research Officer, R&D Centre, Akay Flavours & Aromatics Ltd, Kerala, INDIA, Tel: +91 484 2686111, Fax: +91 484 2680891

Email: [krishnakumar.im@akay-group.com](mailto:krishnakumar.im@akay-group.com)

**Abstract**

Despite the availability of various synthetic drugs for the treatment of functional dyspepsia (FD), their side effects and the cost have always created a great interest in search for novel natural alternatives for the management of gut disorders. The present contribution reports the safety and efficacy of the kitchen spice asafetida (*Ferula asafoetida*) in FD for the first time. In the double-blinded, placebo-controlled study, 43 subjects diagnosed to have moderate to severe discomforts of non-ulcer FD were randomized to receive hard-shell capsules (250 mg × 2/day) of either placebo (n=22) or a food-grade formulation of asafoetida (Asafin) (n=21) for 30 days. When evaluated by a set of validated indexing tools (GSRS, GDSS and NDI), almost 81% in Asafin group showed significant (*p < 0.01*) improvement in the overall score and quality of life as compared to the placebo. At the end of the study, almost 66% of subjects in Asafin group remained symptoms-free. Although the symptoms-score improved significantly in both the groups (from -5.67 to -25.29 in Asafin group *vs*. -1.55 to -6.0 in the placebo; *p ≤ 0.001*), the relative percentage of people who reported more than 80% reduction were in bloating (58 %), appetite (69%), postprandial fullness (74%) motion sickness (75%) and digestion (77%) for Asafin treated group as compared to less than 10% non-specific improvement reported in the placebo group. At the end of the study period, all the subjects remained safe with no adverse events or variations in hematological and biochemical parameters.

[Ethical committee Reg. No: ECR/184/Int/KA/2014; date-15/07/2016, Clinical Trial Reg No: CTRI/ 2018/ 01/011149; date -04/01/2018]

**Figure: S1**

**
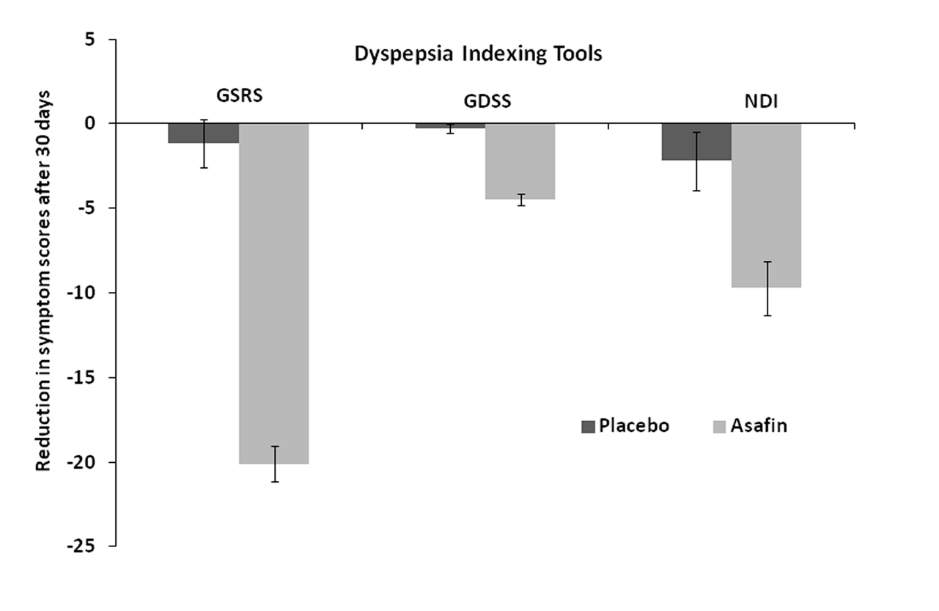
**

**Figure: S1** Reduction in FD Symptom indexing Scores. Values are expressed as Mean ± SEM. The values not sharing a superscript significantly differ at p ≤ 0.001

Table S2

| Symptoms | Odds Ratio | 95% CI Values |
| --- | --- | --- |
| Abdominal pain | 13.6 | 3.09; 59.83 |
| Heart burn | 19.2 | 3.95; 93.38 |
| Bloating | 8.0 | 2.01; 31.80 |
| Constipation | 15.0 | 3.20; 70.39 |
| Borborygmus | 12.0 | 2.62; 54.99 |

Comparison of individual symptom scores as determined by GSRS-Gastrointestinal Symptom Rating Scale, GDSS-Glasgow Dyspepsia Severity Score and NDI-Nepean Dyspepsia Index scales.
